# Development of a Health Text Message System to Support Stroke Prevention: A Component of the Love Your Brain Digital Platform

**DOI:** 10.1111/hex.70471

**Published:** 2025-10-21

**Authors:** Monique F. Kilkenny, Rosanne Freak‐Poli, Catherine Burns, Jan Cameron, Tara Purvis, Mark R. Nelson, Stephanie Ho, Brenda Booth, Janet E. Bray, Lachlan L. Dalli, Eleanor Horton, Timothy Kleinig, Lisa Murphy, Muideen T. Olaiya, Amanda G. Thrift, Seana L. Gall, Dominique A. Cadilhac

**Affiliations:** ^1^ Stroke and Ageing Research, Department of Medicine School of Clinical Sciences Monash University Clayton Australia; ^2^ Stroke and Critical Care Research Theme, Florey Institute of Neuroscience and Mental Health University of Melbourne Heidelberg Australia; ^3^ School of Public Health and Preventive Medicine Monash University Melbourne Australia; ^4^ Menzies Institute for Medical Research University of Tasmania Hobart Australia; ^5^ Stroke Foundation Melbourne Victoria Australia; ^6^ Department of Neurology Royal Adelaide Hospital Adelaide Australia; ^7^ Department of Medicine University of Adelaide Adelaide Australia

**Keywords:** co‐design, consumer engagement, digital health, health communication, mHealth, stroke, text message intervention

## Abstract

**Objectives:**

Globally, stroke is a common cause of death and disability. More than 80% of strokes are reported to be preventable through effective management of modifiable risk factors. Text messages can encourage changes in health behaviour. The Love Your Brain project involves the development and evaluation of a digital health platform for stroke prevention in Australia. In this study, we aimed to develop a text message system and content for this digital platform.

**Method:**

The first phase involved reviewing a repository of existing health promotion messages from prior research on stroke. The second phase included co‐designing the content and delivery of the messaging system with community members (*n* = 12) and health knowledge experts (*n* = 10) through 16 focus groups. New messages were then developed and formatted. These messages were reviewed by subject matter experts, then adjusted for reading age ≤ Grade 10. The final phase included the development of the messaging platform.

**Results:**

Among 1500+ pre‐existing messages reviewed for suitability, ≈10% were adapted for primary prevention. Focus group participants reported that receiving messages on weekdays was preferred and ‘*having a choice’* was beneficial. No consensus was reached regarding message frequency. Weblinks and shorteners were felt to be untrustworthy by participants; therefore, a Love Your Brain website using one hyperlink was developed. New messages were co‐designed and personalised with greetings and sign‐offs to increase engagement. All messages were revised by at least three of eight experts. After editing, 98% were readable at ≤ Grade 10 reading age and 79% at ≤ Grade 8. A REDCap message platform was built to enable personalisation at any time regarding the selection of ‘healthy choices’ relevant to participants' risk factors and preferences for the number of messages per week.

**Conclusion:**

Integrating prior research and co‐design enriched the text message platform, including content and delivery. This system can be adapted for other conditions and cultural needs to deliver relevant health information.

**Patient or Public Contribution:**

People with lived experience of stroke including family/caregivers. and members of the public, actively participated in the co‐design focus groups. The Love Your Brain Management Committee includes people with lived experience of stroke who work in partnership with researchers and clinicians to provide oversight of all stages of the study and the preparation of this manuscript.

## 1 Introduction

Globally, stroke is the third most common cause of death and disability, with 93.8 million prevalent cases of stroke in 2019 [1]. The number of strokes is projected to increase, with predictions that stroke will directly contribute to 300 million disability‐adjusted life years (DALYs), 25 million new strokes and 13 million fatalities by 2050 globally [2]. Importantly, increases in the incidence and prevalence of stroke are occurring among people younger than 65 years [3].

Approximately 80% of strokes are preventable through effective management of modifiable cardiovascular risk factors, such as hypertension, high cholesterol, tobacco smoking, alcohol consumption, diabetes mellitus, physical inactivity and obesity [4]. In the general population, 57% of Australian adults have three or more of these modifiable risk factors [5], with approximately 84% of global stroke DALYs attributable to the most common risk factors [1]. Effectively addressing these risk factors could reduce the burden of stroke, both nationally and internationally [4]. Fortunately, people in the community are interested in seeking knowledge to address their stroke risk. In Australia, the Stroke Foundation's nationwide StrokeSafe Program is a free community education programme about preventing and recognising stroke [6]. Education is delivered in group settings by volunteers who often have lived experience of stroke. Demand for the StrokeSafe Program has increased over time [7], and innovative and low‐cost approaches are needed to deliver stroke education programmes to large populations.

The widespread use of electronic devices in the community, including mobile phones, tablets and computers, can be leveraged to meet the community demand for stroke knowledge and promote health behaviour change. In particular, text messages are gaining popularity as a mechanism to disseminate information to increase awareness of health prevention and engage new audiences [8]. Text message interventions are effective in various areas of health promotion, such as physical activity, diet, weight loss, mental health, smoking and alcohol use [9]. Interventions delivered via text messages hold great potential in preventing cardiovascular disease (CVD) by encouraging a behavioural shift towards healthier lifestyles and medication adherence [10]. An electronic health message system has been developed to support people after stroke [11, 12, 16], but a similar resource does not exist for the prevention of the first stroke.

We aimed to develop a comprehensive text message intervention for primary prevention of stroke that enables tailored messages based on identified risk factors.

## 2 Materials and Methods

### 2.1 Study Design and Participants

The co‐design process was embedded in a Participatory Action Research (PAR) approach, ensuring that community and stakeholder input was central throughout a series of focus groups [13]. It was an iterative process in that discussions from each focus group informed the content of subsequent focus groups. Further details regarding the Love Your Brain co‐design process [14] and recruitment, coordination, participant engagement and satisfaction [15] can be found in prior publications. In brief, the co‐design process involved two separate stakeholder cohorts: health knowledge experts, who were professionals (e.g., neurologist, general practitioner, nurse, allied health practitioner and researcher) involved in health education or health promotion, and adult community members (with or without lived experience of stroke).

### 2.2 Phases for the Development of a Health Text Message System

This intervention was developed as part of Love Your Brain, a digital health platform for stroke prevention which comprises a Massive Open Online Course and text messaging. The content was developed to complement the existing Stroke Foundation StrokeSafe Program by providing additional information and enhancing stroke knowledge retention. The intervention was developed in five phases as outlined below.

### 2.3 Phase 1: Review Existing Messages From Prior Research

We reviewed messages from the Inspiring Virtual Enabled Resources following Vascular Events (iVERVE) system. The iVERVE messaging system is a patient‐centred programme that contains goal‐directed short messages on risk factor management and secondary prevention of stroke [11, 12, 16]. This messaging system has > 1500 messages which were reviewed by multiple topic experts and grouped by an individual's readiness to change and stroke risk factor goal‐setting. Messages were categorised based on the transtheoretical model (TTM) of health behaviour change to time messages about education first (early; contemplation/preparation), followed by motivational messages (late; action/maintenance) [11].

The iVERVE messaging system has been used in a clinical trial, ‘Recovery‐focused Community support to Avoid readmissions and improve Participation after Stroke' (ReCAPS). Participants in the stage II feasibility trial of ReCAPS were satisfied with the content, trustworthiness and delivery of electronic messages to support ongoing recovery and secondary prevention post‐stroke [12, 16].

The text messages used for the ReCAPS trial were provided by the ReCAPS Trial Manager (J.C.). The text messages were entered into Covidence, and two authors (M.F.K. and R.F.) independently reviewed the relevance of each message for primary stroke prevention. Text messages were excluded if they related to stroke recovery or secondary stroke prevention, or were out of scope for the Love Your Brain project. Duplication of messages was independently assessed by two authors (R.F. and C.B.). After removal of duplicates (C.B.), discrepancies about inclusion were discussed in person (M.F.K., R.F. and C.B.), and a pool of the most relevant and appropriate messages for the project were selected. Text messages pertaining to relevant stroke risk factors or messages that could be edited to shift the content from secondary to primary prevention were retained for Phases 2 and 3.

### 2.4 Phase 2: Co‐Design of Messages With Health Knowledge Experts and Community Members

The co‐design process (recruitment, coordination, participant engagement and satisfaction), methods and thematic analysis of the discussions for the broader Love Your Brain project are detailed elsewhere [14, 15]. In brief, the co‐design process involved eight online focus groups, with two separate cohorts of health knowledge experts and other adults from the community. Throughout the co‐design process, participants emphasised the need for simple, easy‐to‐understand language, diverse content, and emotionally engaging material [14]. In this paper, we detail the learnings from the co‐design process specific to the text message intervention.

### 2.5 Phase 3: New Messages Developed and Formatted

Content for the new messages was developed using a combination of existing iVERVE messages, publications, input from co‐design, and information from the Stroke Foundation's website. New messages were required for the topics of primary prevention of stroke, risk factors (e.g., sleep and well‐being) and motivational messages to take action (e.g., encouragement to visit a medical practitioner for cardiovascular risk factor assessment or management). Two authors (R.F. and C.B.) developed new messages, and these were independently reviewed (M.F.K. and J.C.). Commonly accepted abbreviations and plain language were used to ensure a clear understanding by all recipients. ChatGPT (OpenAI, CA) was used to reduce the character count of messages and enhance readability.

Based on similar trials using text message interventions [12, 16], messages were developed so that between 31 and 61 messages could be delivered over 12 weeks. Text messages had to be relevant for people of any age or gender and cater for different education or literacy levels. The maximum message length was 160 characters to enable messages to be sent as a single text message. Previous work has demonstrated that text messages should address participants by name [14]. During the co‐design process (Phase 2), participants discussed signatures (e.g., sign‐off ‘From Love Your Brain’) and the use of weblink shorteners for text messages.

Messages about risk factors were categorised by one author (J.C.) according to the TTM of change [17] and behaviour change techniques [18]. These categorisations were reviewed and confirmed by two authors (R.F. and C.B.).

### 2.6 Phase 4: Expert Review and Refinement

After the development of the text messages, subject matter experts reviewed the content and appropriateness of the messages. Expert reviewers (*n* = 8; expertise in medicine, nursing, nutrition and dietetics, health promotion, health literacy, marketing and communications, and lived experience of stroke) were sourced from Love Your Brain co‐investigators and within professional networks where the co‐investigator group did not cover expertise. Each expert reviewed messages related to their area of expertise (e.g., an expert in nutrition and dietetics reviewed the messages related to healthy eating, achieving and maintaining a healthy weight, and drinking less alcohol; Table 1). After each expert reviewed the messages, feedback was incorporated (R.F. and C.B.) before the messages were sent to the next expert in sequence. Once the final expert review was complete, all messages were assessed for level of readability using an online tool (WebFx Inc., PA; https://www.webfx.com/tools/read-able/) and edited (R.F. and C.B.) for a lower reading level when possible.

**Table 1:** Text message subject matter expert review process.

Order	Expertise	Message topics reviewed
1	Cardiovascular clinical nursing iVERVE and ReCAPS trial staff	All messages
2	Nutrition and dietetics	Healthy eating Achieve and maintain a healthy weight Drink less alcohol
3	Medicine (general practitioner)	Be informed and manage atrial fibrillation Control blood sugar Control blood pressure Control cholesterol
4	Diabetes nursing	Control blood sugar Drink less alcohol Quit and stay smoke‐free Improve well‐being and get enough sleep
5	Health promotion	All messages
6	Health literacy Cardiac care nursing	All messages
7	Person with lived experience of stroke Stakeholder engagement	All messages
8	Marketing and communications	All messages

Abbreviations: iVERVE, Inspiring Virtual Enabled Resources following Vascular Events; ReCAPS, Recovery‐focused Community support to Avoid readmissions and improve Participation after Stroke.

### 2.7 Phase 5: Development of Messaging Platform

A schedule for the delivery of text messages was developed to distribute messages over the 12‐week intervention, utilising feedback from co‐design (Phase 2). We designed the intervention so that all participants would receive the core information messages (*n* = 21) and messages related to one risk factor (*n* = 10) over 12 weeks, with the option to select up to four risk factors (*n* = 10 messages per risk factor). A series of linked databases was developed in REDCap (C.B.) to facilitate the text message platform.

### Statement of Ethics

Ethical approval for this study was received from the Monash University Human Research and Ethics Committee (#35899; 2023). As this was an early development phase research, the testing of the system by researchers was considered not to require written informed consent.

## 3 Results

### 3.1 Phase 1: Review Existing Messages From Prior Research

Of the 1633 messages reviewed, 629 messages were retained for Phase 2 as they were deemed the most appropriate and relevant to this project. There were 368 duplicate messages excluded, with 31 (2%) discrepancies between the two reviewers, of which all messages were retained. Of the 629 messages retained, 19 messages were administrative (e.g., welcome message) and 610 messages were about stroke risk factors.

### 3.2 Phase 2: Co‐Design of Messages With Health Knowledge Experts and Community Members

The co‐design process involved eight online focus groups, for each of the two separate cohorts of health knowledge experts (*n* = 10, clinicians 50%, researchers 50%, 60% ≥ 35 years and 80% female) or other adults from the community (*n* = 12, 58% < 65 years and 58% female). Specifically for text messages, participants highlighted the importance of personalising the experience. For example, only sending text messages about relevant risk factors, and allowing participants to choose the time of day and frequency to receive messages.

Trustworthiness was perceived to be important, with suggestions to send text messages from a consistent email address or phone number, address the recipient by name and sign off messages from ‘Love Your Brain’. Weblinks and shorteners (e.g., abc.ly/Stroke instead of https://strokefoundation.org.au) were reported to be untrustworthy. As described by co‐design participants (Supplementary Table I): ‘*I haven't come across bitly before, so I would not on first contact click on that unless you're prepared to spend some time in the setting it up and teaching people that this is a valid thing (Community)*’, ‘*Personally, I would only click on a URL https: link (Health knowledge expert)’*, ‘*I would not click a link from the text message unless I knew them or very sure it is safe to click (Health knowledge expert)*’. To overcome this hesitancy, we followed the recommendation of a health knowledge expert to use one longer but trusted link in all messages: ‘*Need an alternative if trust is low. It might be better to have a landing page that's short to type in e.g*., www.loveyourbrain.com *and links from there?*’. All links included in the text messages were directed to a project‐specific website (loveyourbrain.org.au) hosted by Stroke Foundation, to enable access to external resources and videos relevant to this project.

### 3.3 Phase 3: New Messages Developed and Formatted

Two sets of messages were designed (21 core information messages and 100 risk factor messages). The core information messages are for all participants. These included an introduction to the study, stroke numbers (burden of stroke), what is stroke (definition), signs of stroke, impact, common risk factors, action plans, and a message to indicate study completion (Table 2). Some of the core information messages were designed to be sent as double messages (i.e., two messages of 160 characters each sent at the same time) to provide vital information at a faster rate at the beginning of the intervention (specifically, messages on the definition and impact of stroke).

**Table 2:** Text Messages for Love Your Brain by stage of change.

	Number of text messages	Stage of change*				
		Pre‐contemplation	Contemplation	Preparation	Action	Maintenance
*n*	121	11	48	4	49	7
%		9%	40%	3%	41%	6%
**Core information**						
Introduction	2					
Stroke numbers	1	1				
What is stroke?	2	1	1			
Signs of stroke	1				1	
Impact of stroke	2	1			1	
Risk factors for stroke	3	2	1			
Action Plan	9		2		7	
Completion	1					
**Healthy choices for risk factors**						
Control blood pressure	10		5		4	1
Start exercising and keep active	10	3		1	6	
Control cholesterol	10		4		4	2
Healthy eating	10	1	3	1	6	
Achieve and maintain a healthy weight	10		3	1	5	1
Quit and stay smoke‐free	10		10			
Be informed and manage atrial fibrillation	10		7		3	
Drink less alcohol	10		5	1	3	1
Control blood sugar	10		5		3	2
Improve well‐being and get enough sleep	10	2	2		6	

* Adapted from the transtheoretical model of health behaviour change [17].

**Figure 1:**
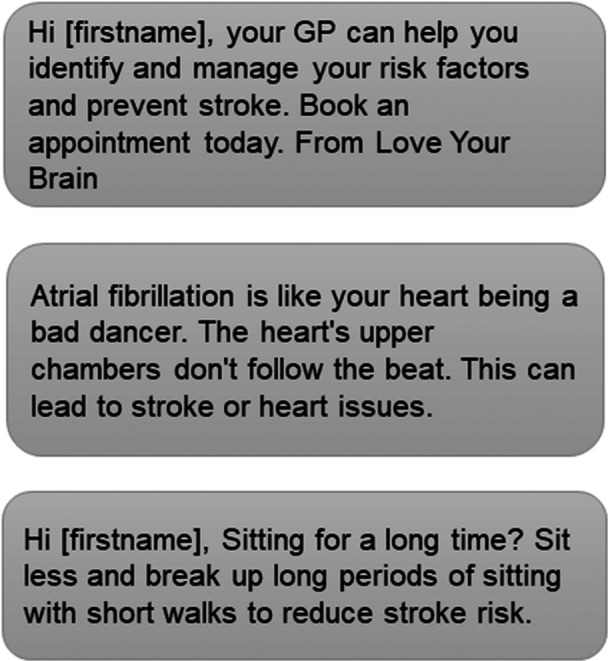
Sample text messages.

The second set of messages related to 10 stroke risk factors. The risk factors were chosen based on population attributable risk [4] and aligned to other public health messaging for stroke prevention, including Life's Simple 7 [19] and Life's Essential 8 [20]. The topics of these messages were: control blood pressure, start exercising and keep active, control cholesterol, healthy eating, achieve and maintain a healthy weight, quit and stay smoke free, be informed and manage atrial fibrillation, drink less alcohol, control blood sugar, and improve well‐being and get enough sleep. For each risk factor, 10 messages were chosen to cover: definition, how the risk factor is related to stroke, number of people affected, signs of the risk factor, treatment/management options, and a call to action (Figure 1).

Based on the TTM [17], 9% of the 121 messages were attributed to the pre‐contemplation stage of change, 40% to contemplation, 3% to preparation, 41% to action and 6% to maintenance (Table 2). In addition, the messages provided prompt intention formation (37%), information about the behaviour health link (31%), instruction (20%) and information on consequences (15%; Supplementary Table II).

To address the feedback and input from the co‐design process, the identifiers of the participant's name and our project name were included. Placeholders for the participant's name ‘Hi <name>’ and a signature ‘Love Your Brain’ were added where character count permitted. The inclusion of emojis was explored, but it was not possible within our selected message distribution platform.

### 3.4 Phase 4: Expert Review and Refinement

Each of the eight experts completed their review within 2–3 weeks, with at least three experts reviewing each message (expertise in medicine, nursing, nutrition and dietetics, health promotion, health literacy, marketing and communications, and lived experience of stroke). Feedback was incorporated, for example, the original message ‘Take out is high in salt = recipe for stroke. For easy and quick home cooking ideas visit <weblink>’ was edited to ‘Takeaway foods high in salt can be a recipe for stroke. For easy and quick home cooking ideas visit <weblink>’.

Of the 121 messages developed, one in five messages were edited to improve readability to a level of Grade 8 and below (equivalent to a reading level of adolescents aged 13–14 years). After finalisation, 84% (102/121) were at a readability level of Grade 8 and below, while 98% (118/121) were at a reading level of Grade 10 and below. When terms such as cholesterol, diabetes and atrial fibrillation were simplified (e.g., ‘diabetes’ changed to ‘high blood sugar’) or the URL removed, all messages achieved a reading level of Grade 8 and below.

### 3.5 Phase 5: Development of Messaging Platform

Phases 1 (iVERVE review) and 2 (co‐design) informed the technical implementation of the text messages. A schedule for delivery of text messages was created to ensure distribution over the 12‐week intervention (Table 3).

**Table 3:** Text message scheduling.

a). Scheduling example for a participant who chooses to receive text messages twice per week (one risk factor)													
	WEEK												TOTAL
	**1**	**2**	**3**	**4**	**5**	**6**	**7**	**8**	**9**	**10**	**11**	**12**	
Introduction	2												**2**
Stroke numbers	1												**1**
What is stroke?	2												**2**
Signs of stroke		1											**1**
Impact of stroke		2											**2**
Risk factors for stroke			1	1		1							**3**
Action plan			1		1		1	1	1	1	1	2	**9**
Control blood pressure		1	1	1	1	1	1	1	1	1	1		**10**
Start exercising and keep active													**0**
Control cholesterol													**0**
Healthy eating													**0**
Achieve and maintain a healthy weight													**0**
Quit and stay smoke‐free													**0**
Be informed and manage atrial fibrillation													**0**
Drink less alcohol													**0**
Control blood sugar													**0**
Improve well‐being and get enough sleep													**0**
Completion												1	**1**
**TOTAL**	**5**	**4**	**3**	**2**	**2**	**2**	**2**	**2**	**2**	**2**	**2**	**3**	**31**

b). Scheduling example of a participant who chooses to receive text messages every weekday (four risk factors)													
	WEEK												TOTAL
	**1**	**2**	**3**	**4**	**5**	**6**	**7**	**8**	**9**	**10**	**11**	**12**	
Introduction	2												**2**
Stroke numbers	1												**1**
What is stroke?	2												**2**
Signs of stroke		1											**1**
Impact of stroke		2											**2**
Risk factors for stroke			1	1		1							**3**
Action plan			1		1		1	1	1	1	1	2	**9**
Control blood pressure		1	1	1	1	1	1	1	1	1	1		**10**
Start exercising and keep active													**0**
Control cholesterol													**0**
Healthy eating		1	1	1	1	1	1	1	1	1	1		**10**
Achieve and maintain a healthy weight													**0**
Quit and stay smoke‐free													**0**
Be informed and manage atrial fibrillation		1	1	1	1	1	1	1	1	1	1		**10**
Drink less alcohol		1	1	1	1	1	1	1	1	1	1		**10**
Control blood sugar													**0**
Improve well‐being and get enough sleep													**0**
Completion												1	**1**
**TOTAL**	**5**	**7**	**6**	**5**	**5**	**5**	**5**	**5**	**5**	**5**	**5**	**3**	**61**

During the baseline survey, each participant will select the healthier choices they want to learn about to manage their identified risk factors. Based on the baseline responses, a minimum of 28 messages to a maximum of 58 messages will be delivered over 12 weeks. Participants will be given the opportunity to change the selection of risk factors, and therefore the number of messages received, at the time of randomisation. The text messages will include:
● Messages relating to core information (including introduction and study completion) sent to all participants (grey shaded in these tables). ● The number of healthier choices the participant chooses in the baseline survey will determine the number of text messages they receive (e.g., participants who choose to learn more about one risk factor receive messages twice per week). Participants will receive messages about at least one risk factor (not shaded), to a maximum of four risk factors ● Risk factors will be ranked on a hierarchy of selection based on the population‐attributable risk [4]. In the instance a participant opts to receive a small number of text messages but selects a higher number of risk factors to learn about, this hierarchy will determine the highest priority risk factors for the participant to receive information about. ● Text messages will contain links to a website (loveyourbrain.org.au) hosted by Stroke Foundation, where resources and videos can be accessed by participants.

The core information messages of introduction, stroke numbers, what is stroke, signs of stroke and impact were given priority in Weeks 1 and 2 to reinforce knowledge imparted in the StrokeSafe programme. The remaining core information messages about common risk factors for stroke, action plan and study completion were delivered over the remaining weeks. For each risk factor selected, one message was delivered each week between Weeks 2 and 11. As a participant could have many risk factors but select only one to four to receive messages about, the allocation of text messages was chosen based on the hierarchy of population‐attributable risk (Table 4) [4], with all participants offered to select risk factors related to physical activity and healthy eating.

**Table 4:** Hierarchy of selection of risk factors.

Rank	Healthy choices for risk factors for stroke
1	Control blood pressure
2	Start exercising and keep active
3	Control cholesterol
4	Healthy eating
5	Achieve and maintain a healthy weight
6	Quit and stay smoke‐free
7	Be informed and manage atrial fibrillation
8	Drink less alcohol
9	Control blood sugar
10	Improve well‐being and get enough sleep

*Source:* Population‐attributable risk factors for stroke, adapted from work published by O'Donnell et al. (2016).

Participants of the co‐design focus groups were unable to reach a consensus regarding message frequency. They expressed preferences for a customisable frequency of messages, with options ranging from daily to weekly, fortnightly or even monthly. Five participants expressed an interest in 2–3 messages per week. For example (Supplementary Table I), ‘*If not able to select individually, would think that 2‐3 per week max (Health knowledge expert)*’ and ‘*Considering it is 12 weeks, I'd say, up to 2 a week would be plenty. I wouldn't mind doing that (Community)’*.

The timing and frequency of messages play a significant role in participant engagement, with concerns raised about feeling bombarded by excessive messaging. Providing participants with the autonomy to select the frequency that suits them best was seen as a favourable approach to maintaining engagement and avoiding message fatigue. Customising the frequency of messages was suggested by 10 participants. For example (Supplementary Table I), ‘*I think having a choice is a good option (Health knowledge expert)*’, ‘*Can participants customize number of messages? (Health knowledge expert)’ and* ‘*I think participants should have a choice of frequency that suits them (Health knowledge expert)*’.

Preferences for receiving messages at specific times of the day were influenced by factors such as work schedules, childcare responsibilities and personal commitments. Participants highlighted the importance of avoiding inconvenient times, such as early mornings or late evenings. Allowing individuals to choose the timing and frequency of messages could improve engagement and adherence to interventions. For example (Supplementary Table I), ‘*Maybe lunchtime or middle of the day…. I'm open to that, but I do think that if you were constantly being bombarded with text early in the morning, you're not going to want to do them (Community)*’, and ‘*Probably also relates to life stage and other commitments. Full time workers may not want mid‐day messages (Health knowledge expert)*’. The consistency of time and day was also highlighted, for example (Supplementary Table I) ‘*Messages received at the same time and same day e.g., 10am Mondays (Health knowledge expert)*’. There was a general consensus that weekdays were preferred to weekend days.

During co‐design (Phase 2), the level of participant choice regarding content and number of messages was highlighted as important for engagement and completion [14]. In addition to the co‐design quotes highlighted previously (Supplementary Table I), participants indicated that: ‘*I think daily runs a risk of becoming intrusive (Community)’*, and ‘*if it was daily, I think that would be an invasion of people's privacy (Community)*’.

**Figure 2:**
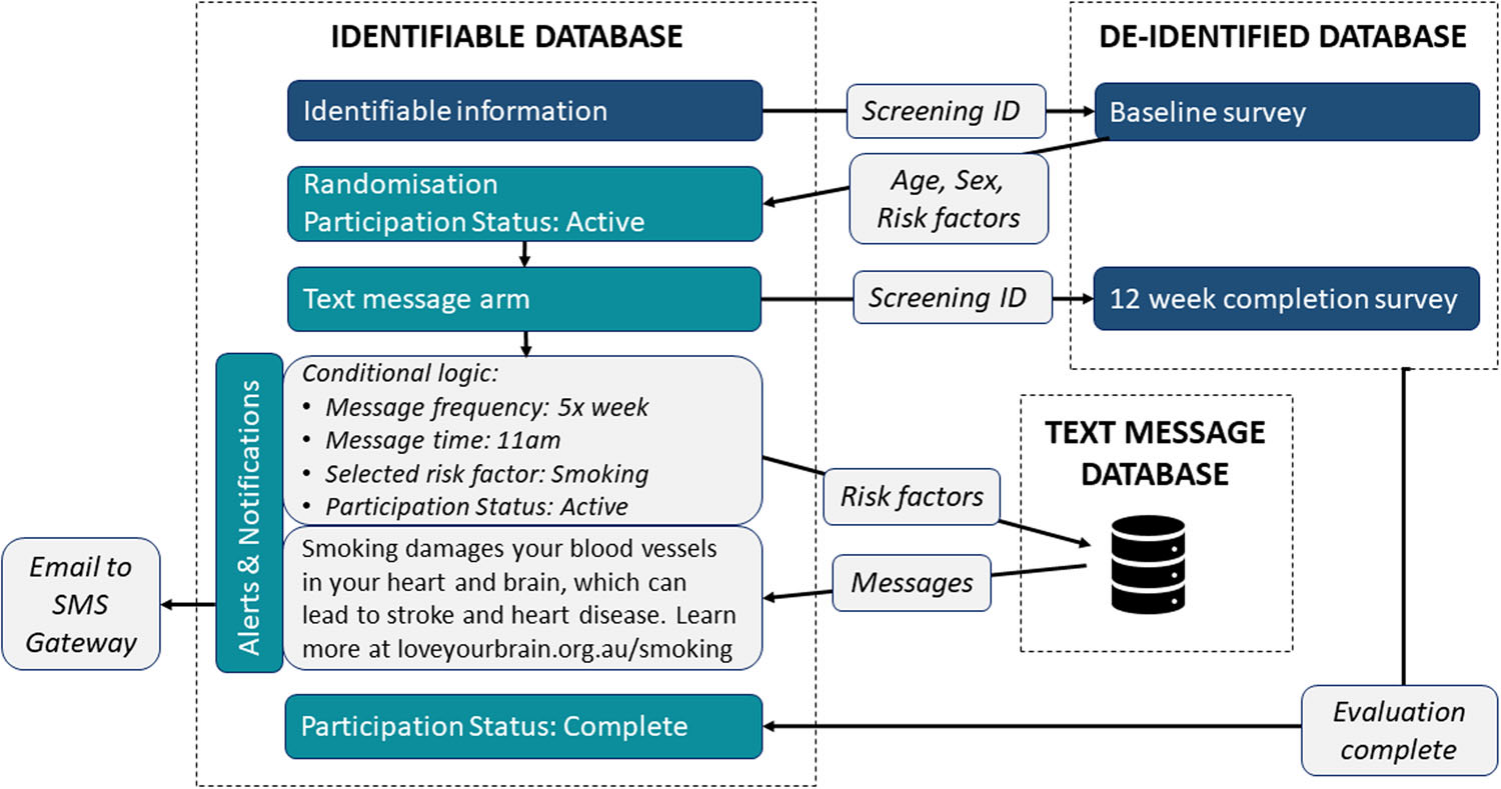
Schematic of the interconnected databases for delivering text messages.

The functionality to send text messages to participants was created within REDCap, hosted by Monash University [21, 22]. A series of three linked REDCap databases were set up with distinct purposes: (i) collect identifiable participant details and facilitate text messaging; (ii) collect de‐identified survey responses; and (iii) a text message repository. Data were mapped between these databases to generate a schedule of notifications for participants in the text message arm, according to their selected risk factors (Figure 2).

Each text message was set up within the REDCap Alerts & Notifications feature. Conditional logic was applied to all alerts, including whether the participant was active in the trial, allocated to the text message arm, and their preferred mode of delivery (SMS or email). For messages about risk factors, additional logic was applied to only send messages related to the topic and the number of risk factors selected. All alerts were scheduled to be sent in sequence from REDCap according to the predetermined schedule, starting from the first Monday following the date of randomisation. All messages were sent at 11 AM Australian Eastern Standard Time. Text messages via email were sent directly from REDCap to the participant, while SMS messages were sent via a third‐party email‐to‐SMS provider using a dedicated phone number. Building trust was emphasised as crucial during the co‐design process, suggesting that using a consistent phone number and email address for messages could facilitate this [14].

We included the functionality in REDCap for participants to change their text message preferences, as suggested during the co‐design stage (Supplementary Table I): ‘*Is there an option that people can ask for more frequent/less frequent message? (Health knowledge expert)*’. The first email sent to participants in the intervention introduced Love Your Brain, outlined their selected risk factors and included a link to change their risk factor selection and, therefore, the number of messages received.

## 4 Discussion

In this study, we outlined the development of a text message intervention for primary stroke prevention and the content and delivery of the text message system. These messages and the system could be expanded for other interventions (e.g., cardiovascular disease more broadly or a specific risk factor such as risky alcohol use). We also highlight the importance of a multifaceted approach incorporating evidence‐based practices and co‐designed insights. By reviewing existing text messages, conducting co‐design focus groups and seeking expert feedback, the intervention was tailored to meet the specific needs and preferences of the target audience. Personalisation was a key outcome of the process, including personalisation of preferred frequency, trustworthy links and content focus (e.g., stroke risk factors).

### 4.1 Delivery and Content

#### 4.1.1 Delivery: Day of the Week

Weekday delivery of interventions, including text message interventions, may result in improved engagement and adherence, compared to weekend delivery. This is supported by results from our focus groups and aligns with previous findings that individuals are more likely to engage with healthier behaviours during weekdays, as weekends often lead to altered routines, sleep patterns, diet and physical activity [23, 24, 25]. Weekend‐heightened health risks are acknowledged as contributors to the phenomenon of the weekend effect, which refers to individuals experiencing poorer health outcomes or higher risks of certain events during weekends than during weekdays [26]. This has been observed in conditions such as heart attack, stroke, suicide and accidents. Other factors include increased stress from social expectations, family obligations or financial concerns; delayed access to healthcare, which is typically reduced on weekends; and fluctuations in mood, emotions or coping strategies. Additionally, human health behaviours tend to follow a circaseptan (7‐day) rhythm due to social and societal factors. This has been demonstrated in analyses of Google search trends, revealing that health‐related search terms are used more frequently on Mondays and Tuesdays, whereas the volume of ‘healthy searches’ declines throughout the week [27]. This trend is also reflected in messages posted to online discussion forums, with a greater number of posts pertaining to health‐related subjects appearing at the start of the week compared to the weekend [28]. In general, people tend to be more motivated to engage in health behaviour changes, such as physical activity, healthy eating and smoking cessation at the start of the week [29, 30]. For example, commencing the intervention on mornings and weekdays, as opposed to afternoons and weekends, had a favourable impact on the utilisation of a free dietary self‐monitoring mobile app [31]. Furthermore, in prior research using the iVERVE system (Stage 1), Wednesdays were the most preferred day for participants to be contacted, followed by Monday and Thursday, while the most preferred time was morning, followed by afternoon, then evening [32].

#### 4.1.2 Delivery: Frequency of Text Messages

Participants prefer to personalise the frequency of text messages. This finding was identified during our co‐design process. Frequency and scheduling were modelled on the digital health programme iVERVE, which was designed to improve self‐management among patients with stroke [11, 12, 16]. Messaging frequency was determined based on how many health goals participants had set for themselves, in addition to motivational and administrative messages. An average of 2 weekly messages per goal was found to be an appropriate frequency by 85% of participants [16]. In qualitative studies of patient experiences with digital apps, participants report a preference to be able to personalise the timing and frequency of messages and prompts [33]. Individualisation of message frequency and timing was also identified as the most efficacious in one meta‐analysis of message‐based health interventions [34]. Optimising text message frequency may help prevent message fatigue, as excessive messaging intensity may lead to users feeling overwhelmed or discouraged [9]. In one pilot study of a weight management digital intervention, most participants reported that four messages per week was an acceptable frequency [35]. These findings, together with our co‐design focus groups, highlight the preference for personalisation of text message frequency, rather than a specific number per week [9].

#### 4.1.3 Content: Personalisation

Personalisation of message frequency and timing, as well as tailoring of message content (e.g., addressing users by their name), has been shown to contribute to improved engagement and motivation [9]. In one systematic review, personalisation or tailoring of the content of mHealth apps to the individual needs of the user was identified as one of the top four positive effects on adherence across all health domains [36]. The use of REDCap as the messaging platform provides flexibility and personalisation, allowing participants to customise their messages based on their individual risk factors and preferences. This feature is essential for promoting long‐term engagement and adherence to the intervention. REDCap has been demonstrated to be a trustworthy and powerful data collection tool for longitudinal studies, including secure encryption, sending study recruitment and invitations, eligibility screening, consenting procedures, lab visit appointments and reminders, data collection confidentiality, and returning test results [37, 38]. REDCap is also appropriate for vulnerable populations, such as among patient‐facing psychological interventions for voice disorders, in which REDCap performed well and effectively in delivering the web‐based intervention [39]. The quality of patient‐reported data collected via REDCap, as well as patients' experiences with the platform, is reportedly positive. In another study, 80.2% of respondents strongly agreed or agreed that the data gathered through REDCap were complete, while 87.9% found the data reliable, and 90.8% considered it accurate [40]. Overall, this indicates that REDCap is an effective, reliable and widely accepted tool for data collection and intervention delivery, praised for its ease of use, customisability, secure data handling and affordability. An additional benefit of REDCap is the potential scalability of the platform to other projects to deliver personalised text messages to participants.

#### 4.1.4 Content: Trustworthiness

The development of a dedicated Love Your Brain website with one link was a strategic decision to address concerns about the trustworthiness of weblinks and shorteners. This approach ensures that participants can easily access additional information related to stroke prevention without leaving the messaging platform. In one systematic review, complementary web access had a positive impact on the adoption and engagement with health and well‐being smartphone apps [41]. This is aligned with a meta‐analysis providing evidence that interventions delivered using the web, compared to non‐web delivery, improved knowledge and behavioural change outcomes [42]. Despite this knowledge, overall use of weblinks is low in mHealth SMS interventions [12]. A benefit of hosting all link content on one website is the ease of checking broken links and updating content. The metrics on clicks and visits to the Love Your Brain website will also be an important part of the process evaluation for the future randomised controlled trial.

### 4.2 Text‐Message Interventions for Stroke Prevention and Cardiovascular Health

Growing evidence highlights the potential of digital health tools to support behaviour change and risk reduction in primary prevention. Broadly, recent literature reviews confirm that text messaging is a promising modality for stroke prevention and cardiovascular health. It is noted in a late‐2023 scoping review of digital stroke interventions that, among various modalities, the strongest evidence of efficacy was for web‐based, telephone and text message programmes [43]. The evidence was particularly strong for improving the stroke risk factors of blood pressure control and medication adherence [43]. Similarly, in a recent systematic review of 22 randomised controlled trials of text‐message interventions targeting CVD prevention, significant improvements in medication adherence and reductions in blood pressure ( ~ 6 mmHg systolic and 2–3 mmHg diastolic compared to controls) were identified [44]. However, no statistically significant changes were observed in other stroke risk factors such as body mass index, cholesterol or blood glucose levels [44].

The benefits of text message interventions have been observed for stroke knowledge among primary and secondary prevention cohorts. A 12‐week randomised controlled trial among Nigerian patients with hypertension and/or diabetes demonstrated that tailored reminder and education texts enhanced awareness of stroke risk factors and prevention practices, even in resource‐limited settings [45]. A motivational text‐message intervention in Scotland to support physical activity after stroke rehabilitation proved highly feasible and well‐received, achieving 100% retention, very high satisfaction and strong engagement [46]. Importantly, participants wanted more personalised tailoring of message content and timing [46], something our co‐designed study directly addresses. However, other programmes have taken personalisation further. For example, in addition to personalisation for time of day, smart watch‐linked messages were timed to contextual factors such as weather in a cardiac rehabilitation trial [47]. These led to immediate increases in step counts within the following hour [47]. However, the effect diminished after 1 month, underscoring the need for ongoing adaptive tailoring to sustain engagement [47]. Furthermore, in a secondary prevention trial for Australian survivors of acute coronary syndrome, there were improvements in body mass index and fruit and vegetable consumption after 12 weeks of personalised texts [48].

Notably, all these examples have resulted in benefits in some, but not all, stroke risk factors. Both the Australian and the Nigerian examples had no statistically significant influence on medication adherence [45, 48]. This may indicate that while knowledge improves, translating this to action across a range of stroke risk factors requires additional support or longer follow‐up. Alternatively, it may highlight that text messaging alone cannot overcome external barriers, such as medication costs or access. Nonetheless, these results reinforce the potential for text message programmes to offer a scalable, low‐cost means of delivering health education and support.

Together, this growing evidence base situates our text‐message intervention within a broader shift towards digital health solutions for stroke and cardiovascular prevention. These examples provide strong evidence that optimising message frequency, timing and content is critical for long‐term efficacy. Our approach—emphasising evidence‐based content, weekday scheduling, personalisation and user co‐design—is consistent with these contemporary findings and directly addresses the challenges of sustaining engagement and reaching at‐risk individuals.

### 4.3 Strengths and Limitations

The study has several strengths. First, our rigorous co‐design process, where structured facilitation and the allocation of adequate resources allowed participants to feel valued and demonstrated high levels of engagement [15]. In turn, the co‐design process ensured that the messages were easy to read, relevant and of interest. Additionally, the messaging platform has flexibility that allows for the personalisation of content and delivery frequency, which could improve adherence to the intervention.

One of the main limitations of this study is that the community sample involved in co‐design had a vested interest in stroke. Our goal was to find a representative sample of our target audience, which consists of people who have not experienced a stroke. However, individuals from the community who were interested in participating either had experience with stroke themselves or were caregivers of someone who had experienced a stroke. Therefore, it is important to test the text message intervention in the target audience during a feasibility study. Another potential limitation is that messages are uniform, and while participants can respond, there is currently no functionality to provide automated responses using generative artificial intelligence technology.

Further developments for this text message intervention could include translation and adaptation for different cultural needs or other health conditions. Additionally, depending on the participant's preference, further refinement of the message bank delivery system may provide further personalisation and specificity.

## 5 Conclusion

The text message intervention developed in this study represents a promising approach to stroke prevention. The co‐design phase, which involved community members and health knowledge experts, ensured that the messages were relevant and tailored to the target audience, increasing the likelihood of engagement. Additionally, the emphasis on readability and the creation of a trusted messaging platform reflects a commitment to health literacy and participant trust. The flexibility built into the platform, allowing for personalisation of content and delivery frequency, caters to individual preferences, potentially enhancing adherence to the intervention. This text message intervention has the potential to reach a wide audience and encourage healthy lifestyle changes. The feasibility trial will provide valuable information on the effectiveness and acceptability of the intervention, paving the way for a larger randomised controlled trial. Upon completion of the trial, the Love Your Brain text message intervention will be disseminated via the National Stroke Foundation (strokefoundation.org.au).

## Author Contributions

**Monique F. Kilkenny:** conceptualization, methodology, writing – original draft, writing – review and editing, funding acquisition, supervision. **Rosanne Freak‐Poli:** conceptualization, methodology, formal analysis, writing – original draft, writing – review and editing, supervision. **Catherine Burns:** conceptualization, methodology, formal analysis, writing – original draft, writing – review and editing. **Jan Cameron:** conceptualization, methodology, formal analysis, writing – review and editing, funding acquisition. **Tara Purvis:** conceptualization, methodology, formal analysis, writing – review and editing, funding acquisition. **Mark R. Nelson:** conceptualization, methodology, writing – review and editing, funding acquisition. **Stephanie Ho:** conceptualization, methodology, writing – review and editing. **Brenda Booth:** conceptualization, methodology, writing – review and editing, funding acquisition. **Janet E. Bray:** conceptualization, methodology, writing – review and editing, funding acquisition. **Lachlan L. Dalli:** conceptualization, methodology, writing – review and editing. **Eleanor Horton:** conceptualization, methodology, writing – review and editing, funding acquisition. **Timothy Kleinig:** conceptualization, methodology, writing – review and editing, funding acquisition. **Lisa Murphy:** conceptualization, methodology, writing – review and editing, funding acquisition. **Muideen T. Olaiya:** conceptualization, methodology, writing – review and editing, funding acquisition. **Amanda G. Thrift:** conceptualization, methodology, writing – review and editing, funding acquisition. **Seana L. Gall:** conceptualization, methodology, writing – review and editing, funding acquisition, supervision. **Dominique A. Cadilhac:** conceptualization, methodology, writing – review and editing, funding acquisition, supervision.

## Ethics Statement

Ethical approval for this study was received from the Monash University Human Research and Ethics Committee (#35899; 2023). As this was an early development phase research, the testing of the system by researchers was considered not to require written informed consent. Universal Trial Number U1111‐1305‐2964.

## Conflicts of Interest

A.G.T. is a previous Board Member of Stroke Foundation; M.F.K. is a member of the Research Advisory Committee of Stroke Foundation; S.L.G. is Chair of Stroke Foundation's Stroke Prevention Advisory Committee and a member of their Clinical Council. M.R.N. reports membership on a Novartis lipids advisory board outside the submitted work. All other authors report no conflicts.

## Supporting information


**Table 1:** Co‐design focus group quotes relevant to text message development. **Table 2:** Text messages categorized by behavior change techniques and illustrative theoretical frameworks.

## Data Availability

The data that support the findings of this study are available from the corresponding author (M.F.K.) upon reasonable request.
