# Supplementary material for: Development of a Health Text Message System to Support Stroke Prevention: A Component of the Love Your Brain Digital Platform
Source: Health Expect. 2025 Oct 21;28(5):e70471. doi: 10.1111/hex.70471 (PMC12539281; doi:10.1111/hex.70471)
Supplement: Supplementary file 1 — Table 1: Co‐design focus group quotes relevant to text message development. Table 2: Text messages categorized by behavior change techniques and illustrative theoretical frameworks. [file HEX-28-e70471-s001.docx]

# Supplemental Material

**Development of a health text message system to support stroke prevention: a component of the Love Your Brain digital platform**

Monique F. Kilkenny, Rosanne Freak-Poli, Catherine Burns, Jan Cameron, Tara Purvis, Mark R. Nelson, Stephanie Ho, Brenda Booth, Janet E. Bray, Lachlan L. Dalli, Eleanor Horton, Timothy Kleinig, Lisa Murphy, Muideen T. Olaiya, Amanda G. Thrift, Seana L. Gall_,_ Dominique A. Cadilhac

**Supplementary Tables**

**Supplementary Table I:** Co-design focus group quotes relevant to text message development

**Supplementary Table II:** Text messages categorized by behavior change techniques and illustrative theoretical frameworks

# Supplementary Table I: Co-design focus group quotes relevant to text message development

| **Focus Group Cohort** | **Mode** | **Quote** |
| --- | --- | --- |
| **Weblinks** |  |  |
| Health Knowledge Expert | Chat | Agree need an alternative if trust is low. It might be better to have a landing page that's short to type in e.g. www.loveyourbrain.com and links from there? |
| Health Knowledge Expert | Verbal | Especially given the audience are likely to be a little bit older, they will be less trusting of these links, and we probably prefer to go onto the website. |
| Community | Verbal | I haven't come across bitly before, so I would not on first contact click on that unless you're prepared to spend some time in the setting it up and teaching people that this is a valid thing. |
| Health Knowledge Expert | Verbal | I would be suspicious of a link. If there was a link I would definitely click on the second one, like the proper URL |
| Health Knowledge Expert | Verbal | I would click the Youtube more than I click the bitly. |
| Health Knowledge Expert | Verbal | I wouldn't trust a link in a message, no matter who it's from, to be honest. If you could maybe say watch to find out, either like click this link or access via the portal wherever it is that they would go on to login independently. |
| Community | Verbal | I'm always a bit reluctant to click on links in text, very cyber aware. Probably a Youtube link with having Youtube been the thing might encourage me a little bit more that I know. It's a little bit safer. |
| Health Knowledge Expert | Chat | Personally I would not click a link from the text message unless I knew them or very sure it is safe to click |
| Health Knowledge Expert | Chat | Personally I would only click on a URL (https: link) |
| Community | Verbal | We're all cyber aware and clicking links is the thing that you're told not to do. |
| Community | Chat | With shortened links you can have tailored ones eg bit.ly/healthyheartcheck |
| **Text message frequency** | |  |
| Health Knowledge Expert | Chat | Agree, 2-3 times max per wek |
| Community | Verbal | Allowing people to choose the frequency and then, having a post participation survey, would allow you to fine tune and have a better understanding of what suits the sample population. |
| Health Knowledge Expert | Verbal | And I think the timing, the frequency people, just feeling they were bombarded was a huge issue. |
| Health Knowledge Expert | Chat | Can participants customise number of messages? |
| Community | Verbal | Considering it is 12 weeks. I'd say, up to 2 a week would be plenty. I wouldn't mind doing that. |
| Community | Chat | Dependent on the person, being 12 weeks, up to 2 per week would be maximum |
| Community | Verbal | Firstly, we have a more frequent series of messages. But as people start to get involved in behavior change like exercise, diet, modification, medication, whatever you might change the messaging. |
| Community | Verbal | For me, If I was doing this I would wanna have it once a week over the 12 weeks |
| Health Knowledge Expert | Verbal | How often do you want your emails to come? And it was really nice daily, weekly, monthly. |
| Health Knowledge Expert | Verbal | I just think to me probably once a week, because you don't want to get them constantly. |
| Community | Chat | I like the weekly approach, on the assumption we’re trying to engage and drive change |
| Community | Chat | I think daily runs a risk of becoming intrusive |
| Health Knowledge Expert | Chat | I think having a choice is a good option. |
| Health Knowledge Expert | Chat | I think participants should have a choice of frequency that suits them. |
| Community | Verbal | I wouldn't like to see more than once one a week, I think. It could become a little bit bothersome |
| Health Knowledge Expert | Chat | If not able to select individually, would think that 2-3 per week max |
| Health Knowledge Expert | Chat | Is there an option that people can ask for more frequent/less frequent message? |
| Community | Chat | Once a month |
| Community | Chat | Once a week |
| Health Knowledge Expert | Chat | Personalize the frequency |
| Health Knowledge Expert | Chat | Personally, more than twice a week, I would start to ignore |
| Health Knowledge Expert | Chat | Setting expectations probably helps but wouldn't want it daily, maybe weekly and would rather choose the day |
| Health Knowledge Expert | Verbal | So one is a high frequency, it is daily and for medium frequencies, every 2-3 days. |
| Community | Verbal | We should just ask people and give them the option of saying I'd like to get them monthly, Weekly Daily Fortnightly, whatever? |
| Community | Chat | Weekly |
| Community | Chat | Weekly |
| Health Knowledge Expert | Chat | Weekly would work for me but its probably variable again |
| Community | Chat | Will people be able to choose the frequency themselves? |
| Health Knowledge Expert | Chat | Would think daily might be too often |
| Community | Verbal | You might do it, as was mentioned, the first couple at the beginning, but if it was daily, I think that would be an invasion of people's privacy. |
| **Text message days and timing** | |  |
| Health Knowledge Expert | Verbal | But it's the timing in the day as well, so if people are at work or they're taking kids to school, you know, they're just gonna ignore it. |
| Health Knowledge Expert | Chat | Could people select when they want to receive the messages when they first sign up? e.g. select day of the week or time period? |
| Community | Verbal | For many would be evening, I think, if they're working and things like that |
| Community | Verbal | Maybe lunchtime middle of the day. I don't know. I'm open to that, but I do think that if you were constantly being bombarded with text early in the morning, you're not going to want to do them. |
| Health Knowledge Expert | Chat | Messages received at the same time and same day e.g. 10am Mondays |
| Community | Chat | Mid morning would work for me, not late in the day |
| Health Knowledge Expert | Chat | Probably also relates to life stage and other commitments. Full time workers may not want mid-day messages. |
| Community | Chat | What about the evening? Younger people working |

# Supplementary Table II: Text messages categorized by behavior change techniques and illustrative theoretical frameworks

| Behavior change technique | **n** | **%** | **Core Information** | | | | | | | | **Healthy choices for risk factors** | | | | | | | | | |
| --- | --- | --- | --- | --- | --- | --- | --- | --- | --- | --- | --- | --- | --- | --- | --- | --- | --- | --- | --- | --- |
|  |  |  | **Welcome** | **Numbers** | **What is stroke?** | **Signs of stroke** | **Impact of stroke** | **Risk factors** | **Action Plan** | **Completion** | **Control blood pressure** | **Control cholesterol** | **Be informed and manage atrial fibrillation** | **Achieve and maintain a healthy weight** | **Control blood sugar** | **Quit and stay smoke free** | **Healthy eating** | **Drink less alcohol** | **Start exercising and keep active** | **Improve wellbeing and get enough sleep** |
| Number of text messages | **121** |  | **2** | **1** | **2** | **1** | **2** | **3** | **9** | **1** | **10** | **10** | **10** | **10** | **10** | **10** | **10** | **10** | **10** | **10** |
| Provide information about behavior health link (IMB) | 37 | 31% |  |  |  |  |  | 1 |  |  | 4 | 4 | 7 | 1 | 5 | 2 | 4 | 1 | 2 | 6 |
| Provide information on consequences (IMB, TRA, TPB, SCogT) | 18 | 15% |  | 1 | 1 |  | 1 | 2 | 1 |  | 5 | 2 | 2 | 1 |  | 2 |  |  |  |  |
| Provide information about others’ approval (IMB, TRA, TRB) | 0 | 0% |  |  |  |  |  |  |  |  |  |  |  |  |  |  |  |  |  |  |
| Prompt intention formation (IMB, TRA, TPB, SCogT) | 44 | 37% |  |  | 1 | 1 |  |  | 4 |  | 1 | 6 | 4 | 2 | 5 | 3 | 4 | 4 | 2 | 7 |
| Prompt barrier identification (SCogT) | 3 | 3% |  |  |  |  |  |  |  |  |  |  |  |  |  | 3 |  |  |  |  |
| Provide general encouragement (SCogT) | 4 | 3% |  |  |  |  |  |  |  |  |  |  |  |  |  | 2 | 1 |  | 1 |  |
| Set graded tasks (SCogT) | 3 | 3% |  |  |  |  |  |  |  |  |  |  |  | 1 |  |  |  | 1 | 1 |  |
| Provide instruction (SCogT) | 24 | 20% |  |  |  |  |  |  | 1 |  |  |  |  | 6 |  |  | 4 | 3 | 7 | 3 |
| Model or demonstrate the behavior (SCogT) | 1 | 1% |  |  |  |  | 1 |  |  |  |  |  |  |  |  |  |  |  |  |  |
| Prompt specific goal setting (CT) | 0 | 0% |  |  |  |  |  |  |  |  |  |  |  |  |  |  |  |  |  |  |
| Prompt review of behavioral goals (CT) | 0 | 0% |  |  |  |  |  |  |  |  |  |  |  |  |  |  |  |  |  |  |
| Prompt self-monitoring of behavior (CT) | 8 | 7% |  |  |  |  |  |  |  |  |  | 1 |  |  | 3 |  | 2 | 2 |  |  |
| Provide feedback on performance (CT) | 0 | 0% |  |  |  |  |  |  |  |  |  |  |  |  |  |  |  |  |  |  |
| Provide contingent rewards (OC) | 0 | 0% |  |  |  |  |  |  |  |  |  |  |  |  |  |  |  |  |  |  |
| Teach to use prompts or cues (OC) | 0 | 0% |  |  |  |  |  |  |  |  |  |  |  |  |  |  |  |  |  |  |
| Agree on behavioral contract (OC) | 5 | 4% |  |  |  |  |  |  | 2 |  |  |  |  | 1 | 1 |  |  | 1 |  |  |
| Prompt practice (OC) | 0 | 0% |  |  |  |  |  |  |  |  |  |  |  |  |  |  |  |  |  |  |
| Use follow-up prompts | 0 | 0% |  |  |  |  |  |  |  |  |  |  |  |  |  |  |  |  |  |  |
| Provide opportunities for social comparison (SCompT) | 3 | 3% |  |  |  |  |  |  | 1 |  |  |  |  |  |  | 1 |  | 1 |  |  |
| Plan social support or social change (social support theories) | 5 | 4% |  |  |  |  |  |  |  |  |  |  |  | 2 | 1 | 1 |  |  | 1 |  |
| Prompt identification as a role model | 0 | 0% |  |  |  |  |  |  |  |  |  |  |  |  |  |  |  |  |  |  |
| Prompt self-talk | 0 | 0% |  |  |  |  |  |  |  |  |  |  |  |  |  |  |  |  |  |  |
| Relapse prevention (relapse prevention therapy) | 0 | 0% |  |  |  |  |  |  |  |  |  |  |  |  |  |  |  |  |  |  |
| Stress management (stress theories) | 0 | 0% |  |  |  |  |  |  |  |  |  |  |  |  |  |  |  |  |  |  |
| Motivational interviewing | 0 | 0% |  |  |  |  |  |  |  |  |  |  |  |  |  |  |  |  |  |  |
| Time management | 0 | 0% |  |  |  |  |  |  |  |  |  |  |  |  |  |  |  |  |  |  |

Behavior change techniques adapted from work published by Abraham and Michie.(Abraham & Michie, 2008)

CT, control theory; IMB, information-motivation-behavioral skills model; MI, motivational interview; OC, operant conditioning; SCogT, social-cognitive theory; TPB, theory of planned behavior; TRA, theory of reasoned action.

Abraham, C., & Michie, S. (2008). A Taxonomy of Behavior Change Techniques Used in Interventions. *Health psychology*, *27*(3), 379-387. <https://doi.org/10.1037/0278-6133.27.3.379>
